# Art and Disease

**Published:** 1894-01-27

**Authors:** 


### Art and Disease.

### III.—MODIFIED FORMS OF POSSESSION.

It has been seen that the phenomena attributed in the present day to hysteria were frequently ascribed in earlier times to the agency of evil spirits. There were cases, however, where, owing to the peculiar character of the symptoms and the sanctity of the possessed, the solution of the mysterious seizure was sought, not in diabolic, but in divine agency.

In certain pictures of the saints in ecstasy, some of the peculair marks of hysteria are plainly perceptible. It is the third phase of the disease, marked by the assumption of fixed attitudes corresponding to some idea present to the patient's mind, which is indicated. The example of Saint Catherine of Sienna must suffice. She is thus described in one of her ecstatic visions: "Her hands and feet were contracted in a convulsive manner, her fingers were interlaced and closed with so much force over the objects she held at the moment of the seizure that they could have been broken sooner than disengaged; all her limbs had the rigidity of stone." In the picture of her by Sodoma, at Sienna, the uplifted hands express the awe and wonder which are reflected in the face. The painter has ignored the painful symptom of rigidity, and has succeeded in rendering the idea of one whirled out of herself by some joyous vision. The desire to render the subject acceptable to the worshippers of the saints has to a great extent modified such paintings and rendered them rather imaginative than real. It is from descriptions such as that cited above in the Lives of the Saints that the close connection between a certain phase of hysteria and a state of ecstasy can be inferred.

But it was not to this phase of serene ecstasy alone that divine inspiration was assigned. During the fourteenth and fifteenth centuries a kind of epidemic raged in the Rhine provinces under the title of the dance of Saint Guy or Saint Vitus (fundamentally differing from the St. Vitus's dance or chorea of to-day), which was regarded with the deepest veneration as a manifestation of special religious fervour. The illustrations of Pierre Breughel, reproduced by Lacroix,* and by M. Charcot,† reveal very clearly indeed the identity of this seizure of spasmodic movement on the part of the pilgrims with hystero-epilepsy. One of these sketches represents "a series of women, each sustained by two men, preceded by players on a sort of bagpipe. They direct their steps, dancing as they go in single file, towards a chapel in the distance containing the relics of the saints. Several of the pilgrims a prey to attacks of which the character cannot be misunderstood, gesticulate, throw themselves into violent contortions, and struggle under the restraint of their companions, who do all in their power to bear them up and hinder them from falling on the ground."

Perhaps no convulsive epidemic presents so many curious features as that which broke out at Paris in the convent of Saint Médard, round the tomb of François de Pâris, the defender of Jansenist doctrines, shortly after his death in 1727. The whole affair is detailed at great length by Carré de Montgeron in a rare and curious volume called "The Truth of the Miracles Worked by the Intercession of M. de Pâris," and profusely illustrated. Certain miracles were early said by the Jansenists to be performed at the tomb of the Abbé, all which miracles were vehemently denied and anathematised by the orthodox party. During the summer of 1731 the simple method of imploring the intercession of the saint at his tomb changed. Those who approached it were thrown into the most violent convulsions. The convulsions became so frequent, and the crowd of "convulsionnaires" so numerous, that an order was given to close the cemetery, while some of the most renowned patients were sent to the Bastille and other prisons. It only wanted this touch of persecution to intensify the manifestations and introduce others even more remarkable. The engravings of the miracles, evidently drawn from life, present many points of pathological interest. Among others, a delineation of the healing of a demoiselle Fourcray, who had been fifteen months deprived of the use of her left foot, illustrates forcibly the connection of these miracles with hysteria. We quote M. Charcot for the details: "The account of the case given enables us to establish that this young lady presented the most varied signs of *la grande névrose*; but the illustration alone is worth far more than a long description. It is sufficient to establish the true nature of the disease, for it is impossible not to recognise at a glance the signs so typical and precise of hysterical club-foot. The demoiselle Fourarcy was then attacked by an hysterical contraction of the left foot. All the interest of this revelation will be realised when it is known that hysterical contraction, which sometimes paralyses a limb for years, far from being incurable, is generally healed in the most unexpected manner suddenly, under the influence of a vivid moral impression, and often at the end of attacks of generalised convulsions."

An engraving representing one of the usual scenes round the tomb of François de Pâris throws further light on the real nature of these "convulsionnaire" seizures. Among crowds of sick folk arriving from all sides several persons are extended in different attitudes expressive of one phase or other of hysterical contraction or convulsion. Some are in the excitement of the "wild movements," while one exhibits a state of complete rigidity. Among the remarkable developments of this epidemic was the ceremony which spread among the convulsionnaires of bearing "aid" to one another. Slight aids consisted in touching, gentle pressure of the hands, &c. The "grand aid," also called by the uncomfortable but not inappropriate name of "the murderous aid," consisted in atrocious acts of violence exerted against each other. Owing to the profound anæsthesia present in those under the influence of an attack, they were able to endure and even invite blows from heavy bars of iron, hammers, &c., and piercing stabs from long nails, needles, and knives. An engraving of Picart's representing one of these grand aids represents the patients in the act of being trampled on by several of those assisting, and it is worthy of remark that M. Charcot has recorded the curious efficacy of pressure on certain zones of the body in many cases of hysteria.

* "Vie Militaire et Religieuse au Moyen Age," page 439.

† "Maladie du Système Nerveux," vol. II., page 456.

